# Efficacy of Oxygen-Enriched Mouthwash as a Pre-procedural Mouth Rinse Against Oral Microbes Produced During Ultrasonic Scaling

**DOI:** 10.7759/cureus.49164

**Published:** 2023-11-21

**Authors:** Vyshnavi B Sindhusha, Arvina Rajasekar

**Affiliations:** 1 Periodontics, Saveetha Dental College and Hospitals, Saveetha Institute of Medical and Technical Sciences, Saveetha University, Chennai, IND

**Keywords:** infection prevention and control, chlorhexidine gluconate mouthwash, blue m, pre-procedural rinse, aerosol contamination

## Abstract

Aim

Oxygen-enriched mouthwash products are based on oral topical oxygen therapy (OOT), which supports the formation of new blood vessels and the removal of toxins and waste products from the affected area and stimulates the synthesis of collagen. These antioxidant mouthwashes contain honey, lactoferrin, and sodium carbonate peroxide. Lactoferrin is an anti-inflammatory protein that binds the ferrous iron ions surrounding micro-organisms regulating bacterial growth. Hence, these products can be included as an adjunct to toothbrushing after oral surgeries and in the treatment of conditions like gingival inflammation and peri-implantitis. The aim of the study was to evaluate the efficacy of oxygen-enriched mouthwash as a pre-procedural mouth rinse against oral microbes in the aerosol produced during ultrasonic scaling.

Materials and methods

A total of 40 patients with an age range of 20-40 years who have been advised to undergo ultrasonic scaling were selected as study subjects and were randomly allocated to group 1 (test group; n = 20; blue®m mouthwash) and group 2 (control group; n = 20; chlorhexidine). After evaluating the initial bacterial load after the use of water (placebo) as pre-rinse on the patient's chest and shoulder areas in both experimental and control groups, both the group subjects were instructed to gargle with 10 ml of the provided mouth rinse for one minute before ultrasonic scaling procedure. Blood agar plates were placed at the patient's chest and shoulder area to collect the aerosol and were later incubated to assess the colony-forming units (CFUs). An independent t-test was done to compare the CFUs between the groups.

Results

The mean initial bacterial load after the use of water (placebo) as pre-rinse on the patients' chest area (122.4 ± 0.6) and shoulder area (109.3 ± 2.6) in the experimental group was similar to the bacterial load seen on the chest area (126.2 ± 4.8) and shoulder area (115.4 ± 3.8) in the control group. The CFUs found in blood agar plates placed on the chest (59.8 ± 2.5) and shoulder (35.3 ± 3.6) areas of patients in group 1 were less as compared to CFUs found in blood agar plates placed on the chest (104.8 ± 3.2) and shoulder (75.3 ± 2.8) areas of patients in group 2. The difference between both groups was statistically significant with a p-value of ≤0.05.

Conclusion

There is a reduction in the bacterial load in the aerosols that are emitted during the ultrasonic scaling procedure with the use of oxygen-enriched mouthwash as a pre-procedural rinse when compared with chlorhexidine.

## Introduction

Dentists and dental personnel work in a highly contaminated environment. This contamination poses a potential risk of infection. Dental procedures, such as drilling, scaling, and other treatments can generate aerosols and splatter [1]. Aerosols are fine, suspended particles in gas form with sizes typically less than 50 μm [2], while splatter consists of larger airborne particles with sizes greater than 50 μm. Aerosols may contain oral microorganisms, saliva, blood, and other debris from the teeth, which could cause potential infections like tuberculosis (TB), which is primarily transmitted through droplets. Also, patients with compromised immune systems are particularly vulnerable to infections transmitted through aerosols [3]. Proper sterilization and disinfection of dental instruments and equipment are critical to prevent the transmission of such infections. Following the American Dental Association (ADA) recommendations for instrument sterilization, the treatment of dental unit waterlines (DUWLs) with regular flushing and disinfection of DUWLs is a critical step in controlling major sources of potentially contaminated dental aerosols [4].

Chlorhexidine gluconate is highly effective as a pre-procedural rinse for infection control and reducing the potential transmission of pathogens through dental aerosols. Studies have shown that using 0.12% chlorhexidine as a pre-rinse 10 minutes before air polishing results in a greater reduction in airborne microbial load [5]. Pre-procedural rinsing with an antiseptic mouthwash containing chlorhexidine gluconate led to a substantial (94.1%) reduction in microbial colony-forming units (CFUs) [6].

Despite its efficacy, chlorhexidine (CHX) does have some potential adverse effects, including dry mouth, desquamation of the oral mucosa, swelling of the parotid gland, and oral paresthesia. Tooth staining is one of the most undesirable side effects of chlorhexidine, which occurs due to the formation of pigmented metal sulfides on the tooth surface [7].

Oxygen-enriched mouthwash (blue®m) utilizes oxygen as an active ingredient. Oxygen has been found to play a crucial role in various stages of the healing process, including re-epithelialization, angiogenesis, and collagen synthesis [8]. Oxygen therapy aims to expedite healing by promoting neovascularization, removing toxins, stimulating the formation of new blood cells and stem cells, and eliminating bacteria [9]. The use of antioxidant mouthwash can be a viable alternative to CHX in postsurgical care, as it does not exhibit the disadvantages associated with CHX, particularly its cytotoxic effects on gingival cells [10]. The oxygen-enriched mouthwash consists of sodium carbonate peroxide along with honey, xylitol, and lactoferrin, which makes it useful in the treatment of inflammation in the gums along with other conditions such as peri-implantitis [11]. This study was conducted to assess the efficacy of oxygen-enriched mouthwash (blue®m) as a pre-procedural rinse during ultrasonic scaling.

## Materials and methods

**Figure 1:**
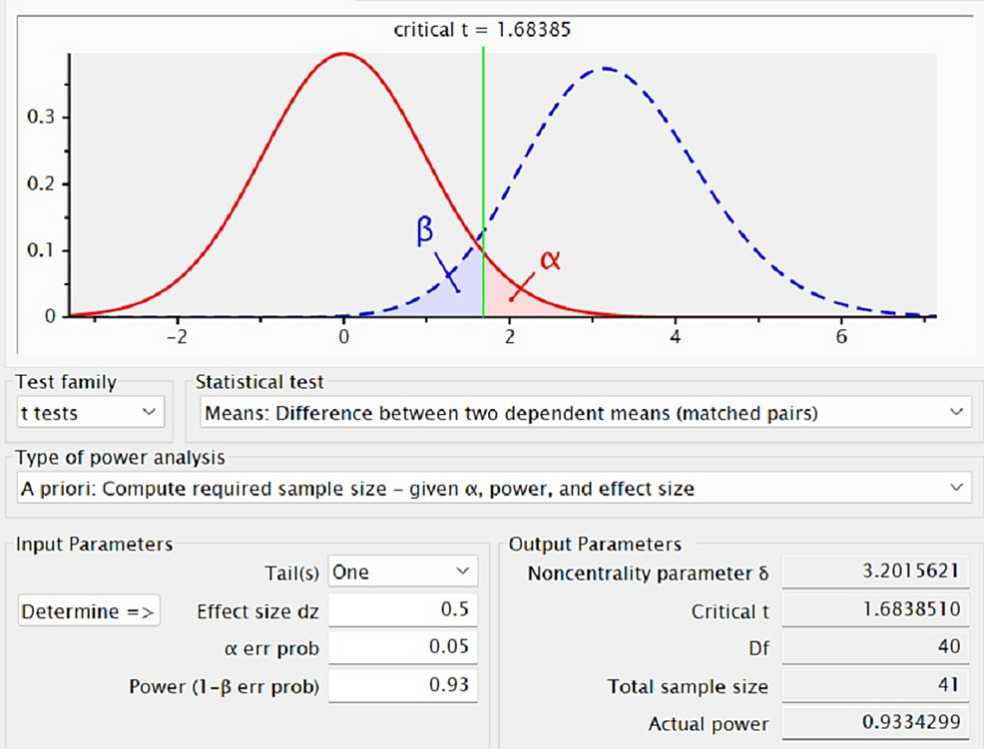
Sample size calculation using G*Power software

The present clinical study was conducted among 40 patients who had undergone ultrasonic scaling in the Department of Periodontics, Saveetha Dental College and Hospitals, Chennai. Study protocol approval was obtained from the Human Ethics Committee of Saveetha Dental College and Hospitals (IHEC/SDC/PERIO-2104/22/234). Also, consent was obtained from all the study participants prior to the start of the study. The sample size was calculated using the data from the previous study [12] using G*Power software (Figure 1).

Inclusion criteria

The study included subjects aged 20-40 years with gingival index (Loe & Silness gingival index, 1960) and plaque index (Silness & Loe plaque index, 1963) scoring values between 1.1-2.0 and 1.0-1.9, respectively. All the subjects with at least 20 healthy teeth were included in the study.

Exclusion criteria

Periodontitis patients and patients who had undergone periodontal treatment within the last six months, patients with a known history of systemic diseases and smokers, pregnant and nursing mothers, and patients who were under long-term antibiotics or anti-inflammatory drugs were excluded from the study.

Methodology

Patients who reported to the Department of Periodontics in Saveetha Dental College and Hospitals from April 2023 to June 2023 were randomly allocated to group 1 (test group; n = 20) and group 2 (control group; n = 20). The initial bacterial load of both groups was evaluated after the use of water (placebo) as a pre-rinse followed by ultrasonic scaling. Subjects in group 1 were given 10 ml of blue®m mouthwash (developed by a team of dental surgeons led by Dr. Peter Blijdorp in the Netherlands) and 10 ml of chlorhexidine mouthwash (0.12% concentration, Hexidine, ICPA Health, Ankleshwar, India) was given to subjects in group 2 as a pre-procedural rinse before ultrasonic scaling. Both the group subjects were instructed to gargle with the provided mouth rinse for one minute before the scaling procedure.

To capture the microorganisms released from aerosols during scaling, blood agar plates were used, as they are a reliable medium for cultivating bacteria. These plates were placed on the patient's chest and shoulder area. At the time of scaling, low-volume saliva ejectors were used and the patient's mouth was at a distance of 6 inches from the blood agar plates. The same practitioner performed full mouth ultrasonic scaling on all patients (VS) and the scaling duration for each patient was around 15-20 minutes. After the scaling was completed, the blood agar plates were left in place for 10 minutes to allow aerosols to settle.

Following the scaling procedure, the agar plates were incubated at 37°C for 24 hours in the incubator. CFUs in the agar plates were then counted and statistical analysis was performed on the collected data.

Statistical analysis

Data collected were statistically analysed using SPSS version 20 software (IBM Corp., Armonk, NY). An independent t-test was done to compare the CFUs found on blood agar plates placed on the chest and shoulder areas of groups 1 and 2. The difference between both groups was statistically significant with a p-value of ≤0.05.

## Results

**Figure 2:**
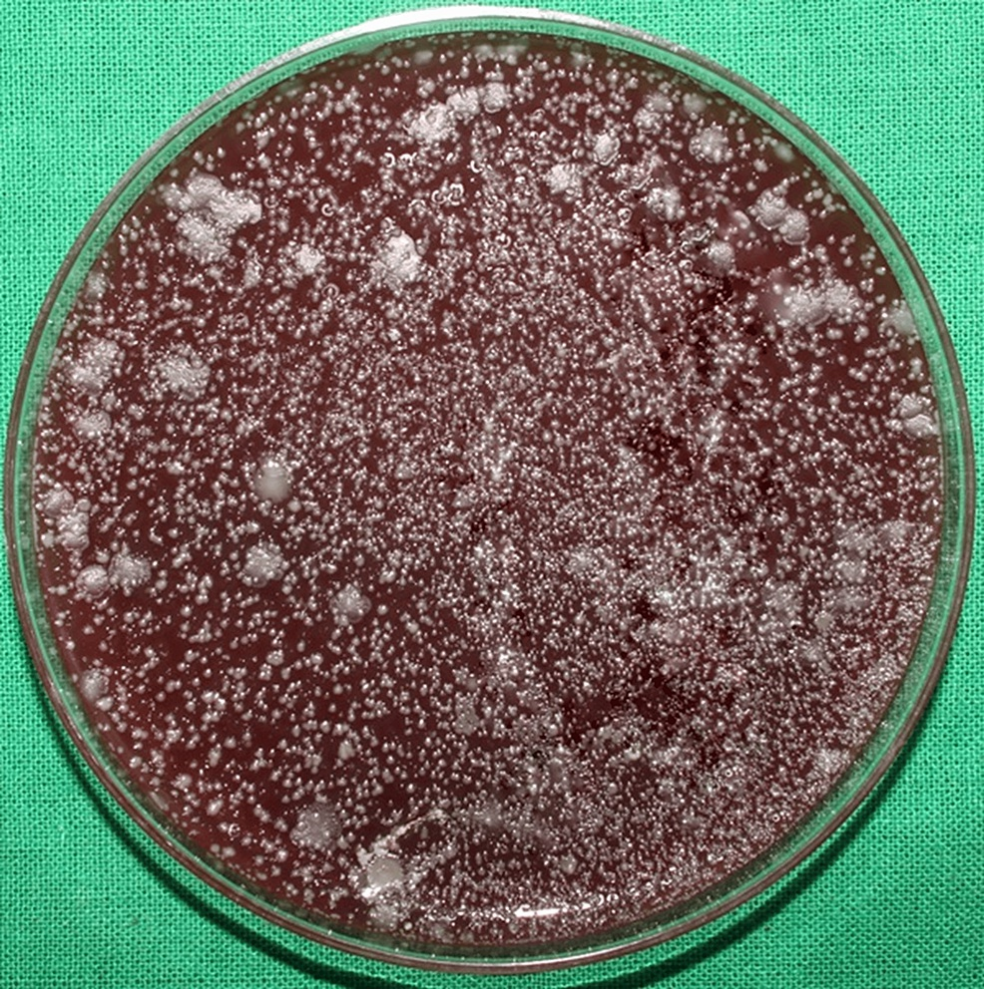
Initial bacterial load on the patient's chest area in the experimental group The image shows the colony-forming units (CFUs) on the blood agar plates placed on the patient's chest area after the use of water (placebo) as a pre-rinse indicating the initial bacterial load of the experimental group.

**Figure 3:**
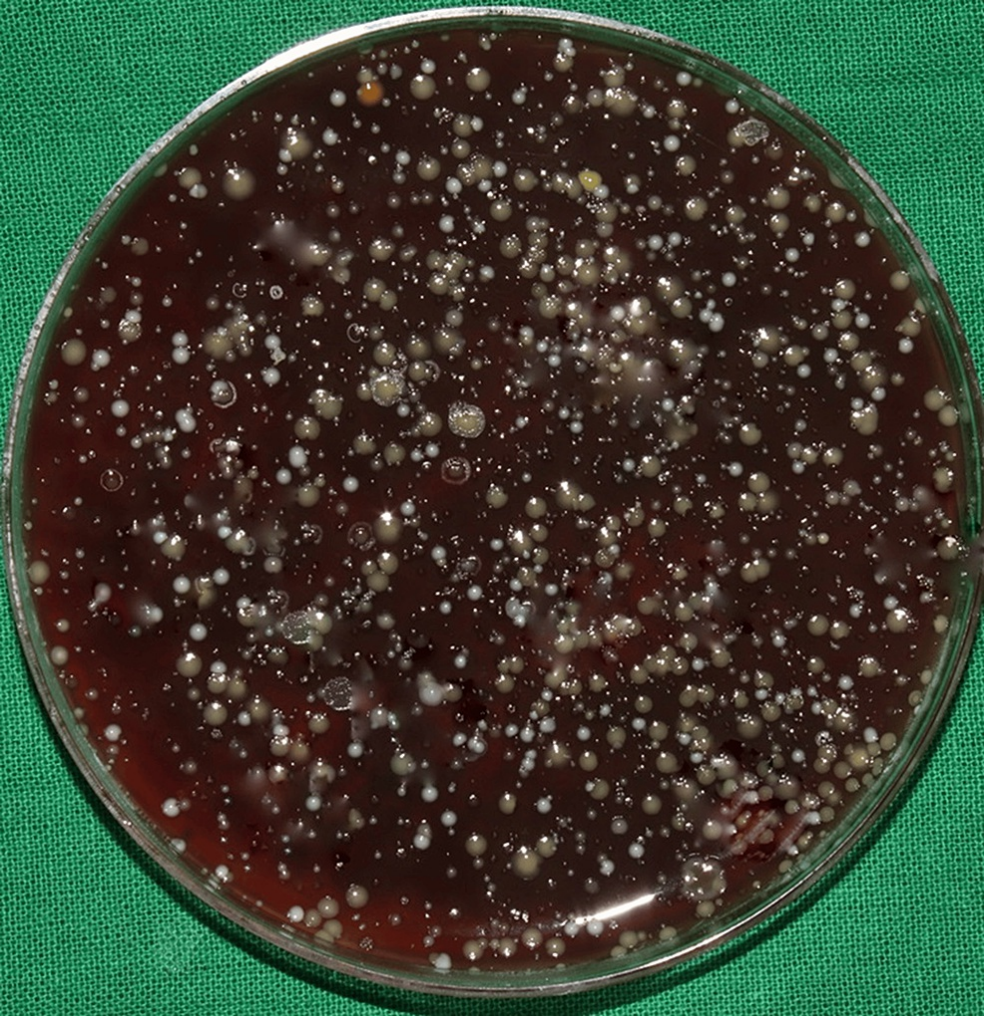
Initial bacterial load on the patient's shoulder area in the experimental group The image shows the colony-forming units (CFUs) on the blood agar plates placed on the patient's shoulder area after the use of water (placebo) as a pre-rinse indicating the initial bacterial load of the experimental group.

Among the experimental group patients, the initial bacterial load after using water (placebo) as a pre-rinse followed by ultrasonic scaling with the CFUs formed on the blood agar plate in the chest and shoulder areas is illustrated in Figures 2, 3, respectively.

**Figure 4:**
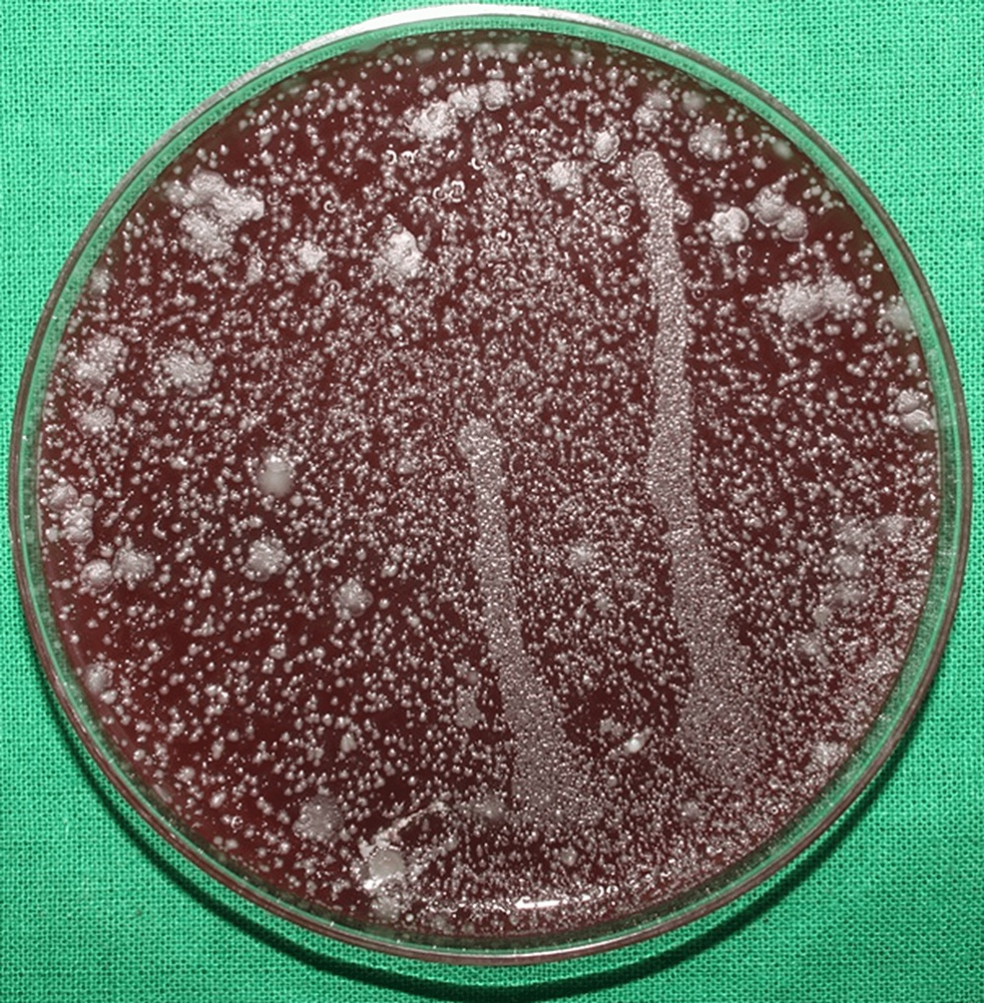
Initial bacterial load on the patient's chest area in the control group The image shows the colony-forming units (CFUs) on the blood agar plates placed on the patient's chest area after the use of water (placebo) as a pre-rinse indicating the initial bacterial load of the control group.

**Figure 5:**
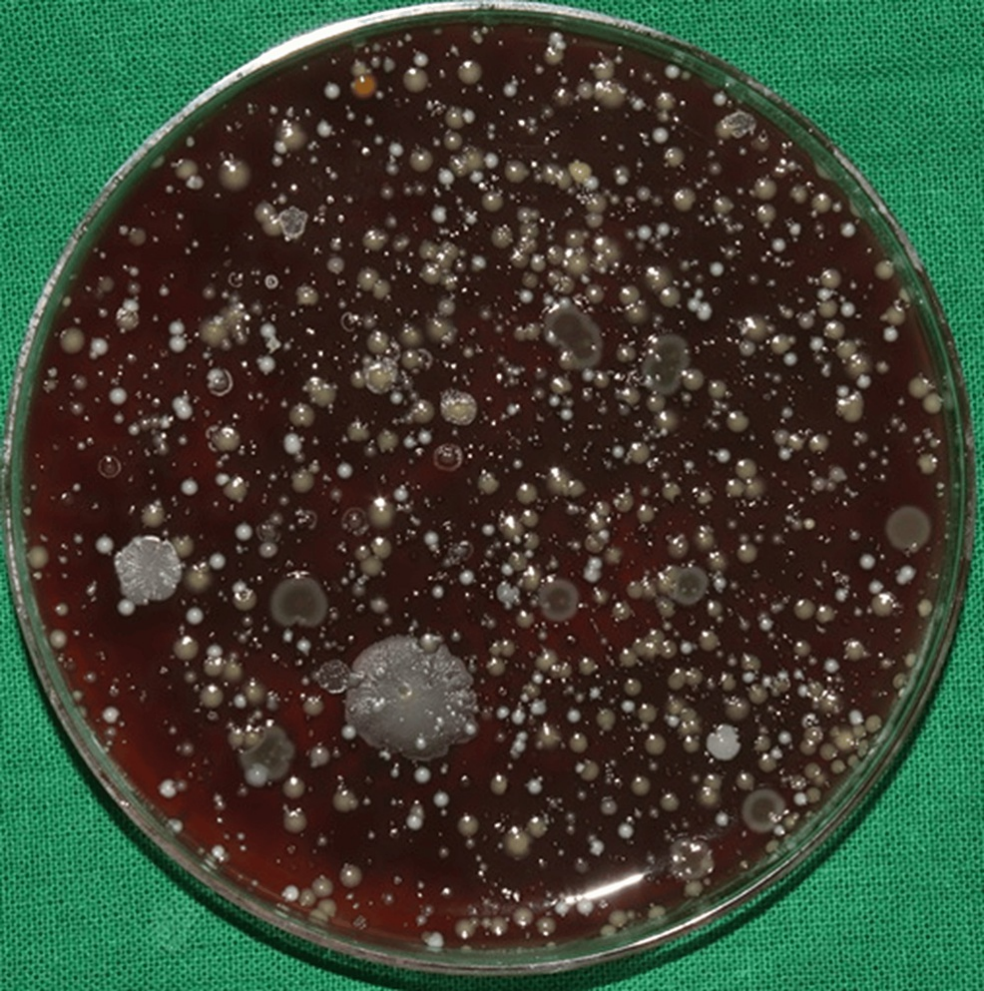
Initial bacterial load on the patient's shoulder area in the control group The image shows the colony-forming units (CFUs) on the blood agar plates placed on the patient's shoulder area after the use of water (placebo) as a pre-rinse indicating the initial bacterial load of the control group.

Among the control group patients, the initial bacterial load with the CFUs formed on the blood agar plate in the chest and shoulder areas is illustrated in Figures 4, 5, respectively.

**Figure 6:**
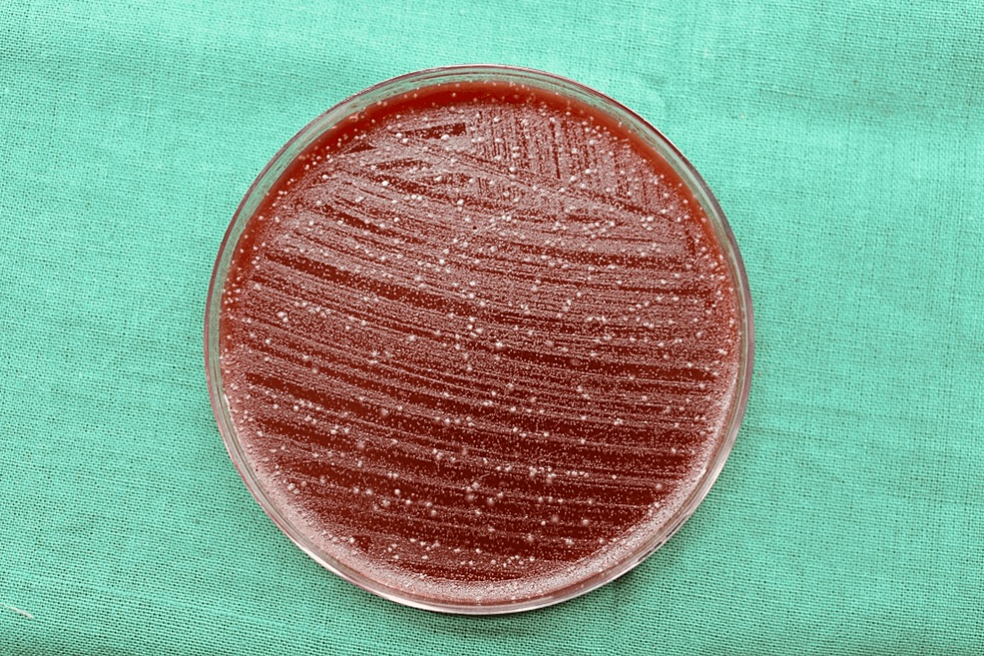
CFUs on the blood agar plate placed on the patient’s chest area after the pre-procedural rinse in the experimental group Colony-forming units (CFUs) on the blood agar plate placed on the chest area of the experimental group where blue®m mouthwash was used as a pre-procedural rinse.

**Figure 7:**
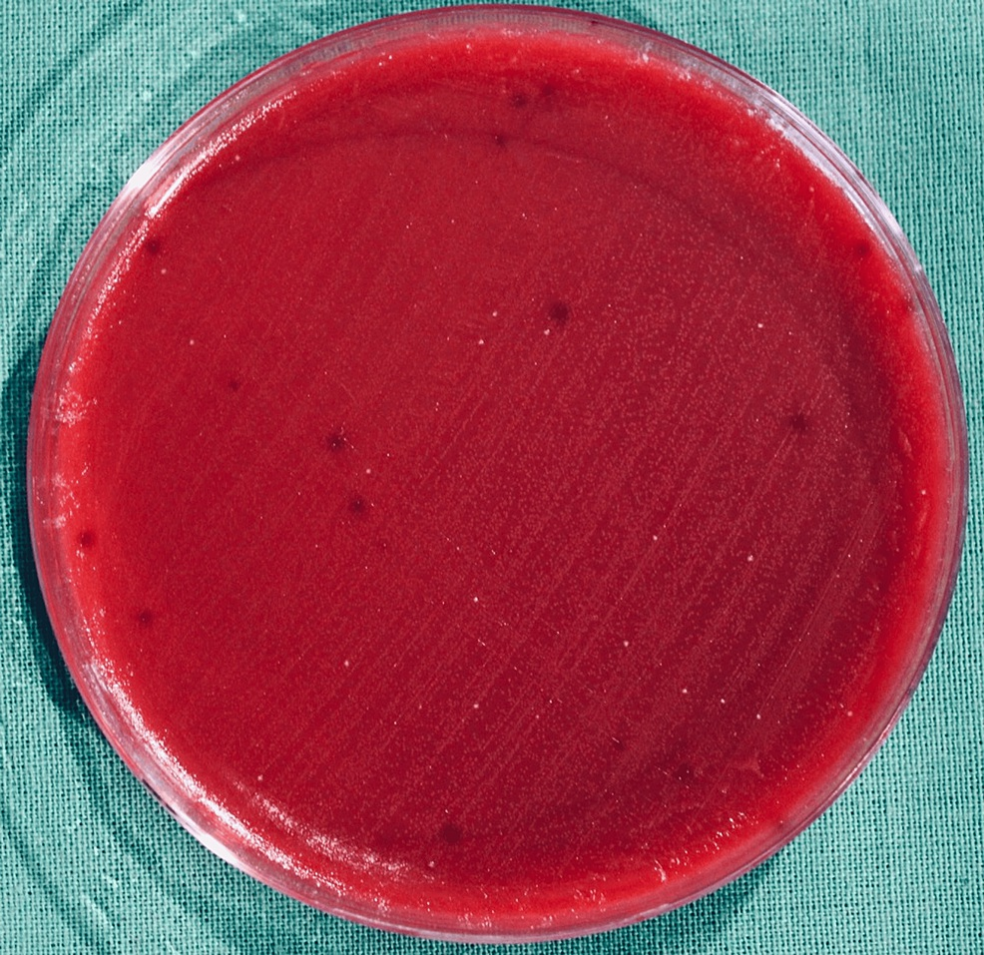
CFUs on the blood agar plate placed on the patient’s shoulder area after the pre-procedural rinse in the experimental group Colony-forming units (CFUs) on the blood agar plate placed on the shoulder area of the test group where blue®m mouthwash was used as a pre-procedural rinse.

Among the experimental group patients, the CFUs formed on the blood agar plate in the chest and shoulder areas are illustrated in Figures 6, 7, respectively.

**Figure 8:**
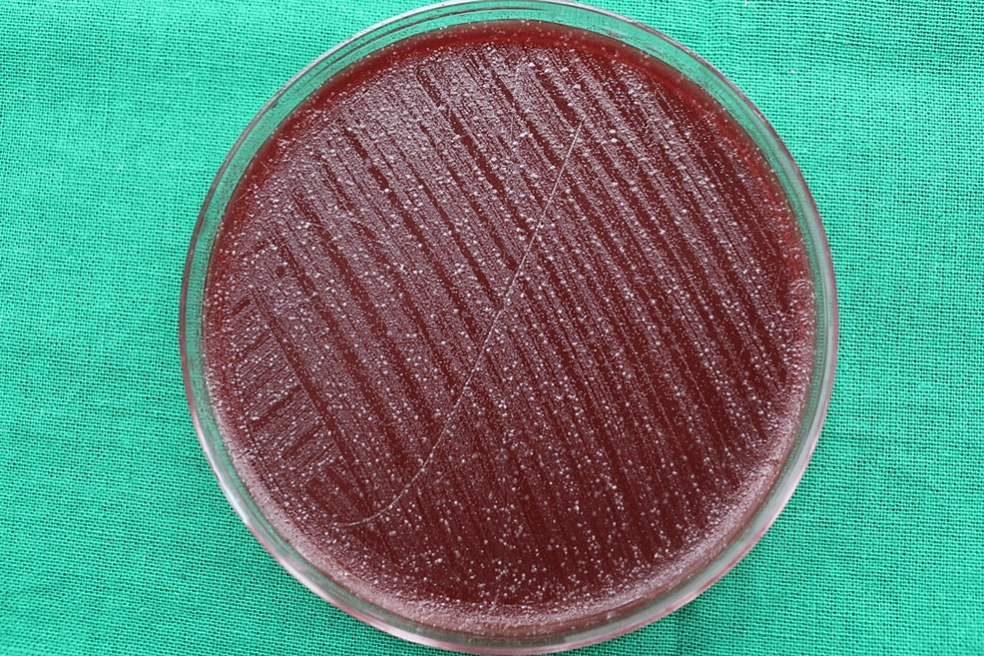
CFUs on the blood agar plate placed on the patient’s chest area after the pre-procedural rinse in the control group Colony-forming units (CFUs) found on the blood agar plate placed on the patient's chest area where chlorhexidine mouthwash was used as a pre-procedural rinse.

**Figure 9:**
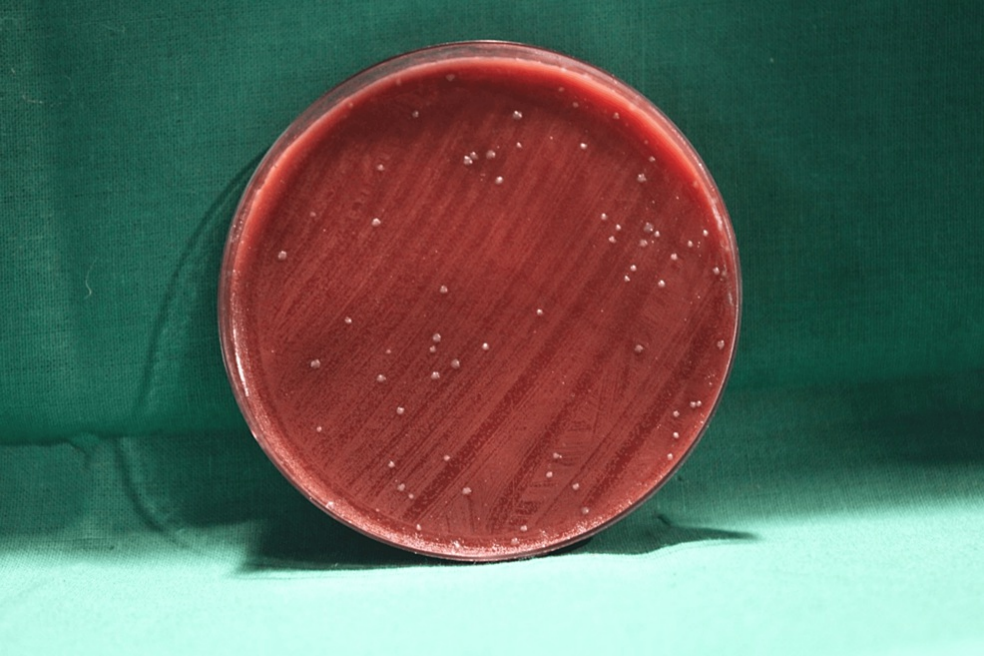
CFUs on the blood agar plate placed on the patient’s shoulder area after the pre-procedural rinse in the control group Colony-forming units (CFUs) found on the blood agar plate placed on the patient's shoulder area where chlorhexidine mouthwash was used as a pre-procedural rinse.

Among the control group patients, the CFUs formed on the blood agar plate in the chest and shoulder areas are illustrated in Figures 8, 9, respectively.

The mean and standard deviation of the CFUs on blood agar plates based on the location of agar plates during the scaling procedure are given in Table 1.

**Table 1:** Mean and standard deviation of the CFUs This table shows the values of the mean and standard deviation (SD) of the colony-forming units (CFUs) in the blood agar plates based on the location of the agar plates. * P-value is less than 0.05. The post-procedural rinse between the experimental and control groups is statistically significant.

Group wise comparison	The chest area of the patients	The shoulder area of the patients
Initial bacterial load in the experimental group (mean ± SD)	122.4 ± 0.6	109.3 ± 2.6
Initial bacterial load in the control group (mean ± SD)	126.2 ± 4.8	115.4 ± 3.8
P-value	0.19	0.12
Post-treatment bacterial load in the experimental group (mean ± SD)	59.8 ± 2.5	35.3 ± 3.6
Post-treatment bacterial load in the control group (mean ± SD)	104.8 ± 3.2	75.3 ± 2.8
P-value	0.03*	0.02*

The results show that the initial bacterial load on patients' chest area (122.4 ± 0.6) and shoulder area (109.3 ± 2.6) after the use of water (placebo) as pre-rinse in the experimental group was statistically insignificant (p > 0.05) when compared with the initial bacterial load on patients' chest area (126.2 ± 4.8) and shoulder area (115.4 ± 3.8) in the control group. Whereas after pre-procedural rinsing with respective mouthwashes, the CFUs found in blood agar plates placed on the chest (59.8 ± 2.5) and shoulder areas (35.3 ± 3.6) in the patients of the experimental group were lesser as compared to the CFUs found in blood agar plates placed on the chest (104.8 ± 3.2) and shoulder (75.3 ± 2.8) areas of the patients of the control group. This difference in the CFUs between the groups post and pre-procedural rinse was statistically significant with a p-value ≤ 0.05.

## Discussion

Ultrasonic scalers are effective in removing plaque and calculus by disrupting the plaque biofilm. This can release a high concentration of bacteria and viruses into aerosols, which poses potential risks of disease transmission among patients and dental healthcare providers [13]. To address this, infection control measures are taken, which mainly include the usage of high-volume evacuation (HVE) systems, which are designed to capture and remove aerosols and disperse airborne particles [14]. Also, proper disinfection protocols should be followed rigorously to prevent bacterial contamination from aerosols [15].

It is proven that using pre-procedural rinses before treatment helps to reduce the microbial load in the patient's oral cavity, thereby minimizing the bacterial load in aerosols. CHX is considered one of the most effective options for lowering aerosol contamination [16]. However, it has a cytotoxic effect on human gingival fibroblast cells, periodontal ligament cells, gingival epithelial cells, and osteoblastic cells. Also, it causes a few side effects, which include teeth staining, taste alteration, and parotid gland swelling [17]. Hence, some of the herbal extracts/natural compounds and antioxidants have been investigated for their ability to inhibit the growth of oral bacteria. These alternatives to the systemic antimicrobials are being explored as potential components of pre-procedural mouth rinses as well [18].

Literature evidence reveals that various commercially available mouthwashes contain antibacterial chemicals, such as chlorhexidine and cetylpyridinium chloride, which are cytotoxic to the oral tissue, while the oxygen-enriched mouthwash releases active oxygen when it comes in contact with the oral tissues that help in the elimination of bacteria and whiten teeth and has a sanitizing effect [19]. blue®m mouthwash contains honey, lactoferrin, and sodium carbonate peroxide. Honey is an active ingredient of the mouthwash, as it causes glucose oxidation in the oral cavity that releases active oxygen. This active oxygen helps in osmosis of bacterial cells leading to bacterial dehydration, thus exhibiting antimicrobial action. It also contains lactoferrin, which is an anti-inflammatory protein that binds to the ferrous iron ions surrounding the microorganisms. The sodium carbonate peroxide present in the product releases oxygen when it comes in contact with oral tissues, thus making the tissues less conducive to the growth of anaerobic bacteria. Also, they are known for their ability to remove surface stains from teeth, leading to improved aesthetics [20]. In this context, this research compared the efficacy of blue®m with the conventional CHX as a pre-procedural rinse among patients undergoing ultrasonic scaling. This is the first study of its kind to assess the efficacy of blue®m as a pre-procedural mouth rinse.

The present study data showed that there was a lesser microbial colony count in the aerosols among the patients who had undergone pre-procedural rinse using the blue®m mouth rinse as compared to CHX. As this is the first study using blue®m as a pre-procedural mouth rinse, a direct comparison of the present study findings was not possible. However, studies that assessed the antimicrobial effect of blue®m were considered for comparison.

Cunha et al. demonstrated that toothpaste containing active oxygen and lactoferrin has comparable antiplaque and anti-gingivitis efficacy with triclosan-containing toothpaste [21]. Similarly, when Shibli et al. compared two different antimicrobial agents, a hydro-carbon-oxo-borate complex, and chlorhexidine in the context of subgingival biofilm formation and their effects on bacterial species, it was observed that the hydro-carbon-oxo-borate complex has more antimicrobial action, especially in reducing red complex pathogens when compared to chlorhexidine. The antimicrobial effect of the hydro-carbon-oxo-borate complex is due to the chemical formulation of the peroxoborate complex, which releases reactive oxygen species (ROS) that exhibit antibacterial properties. Also, it increases the level of oxygen in tissues, thereby improving healing [22].

In addition, blue®m has low cytotoxicity and high compatibility. Also, it modulates the gene expression of *Streptococcus mutans*, proving to be a good therapeutic plaque control agent [23]. Furthermore, blue®m was found to be a safe alternative to CHX in reducing the total microbial colony counts in plaque samples obtained from gingivitis patients. The authors suggested that blue®m was equally effective in reducing the microbes when compared to CHX [24].

Furthermore, when the antimicrobial efficacy of the blue®m mouthwash was tested against chlorhexidine mouthwash in dental implant patients using the plaque sample taken pre and post-implant surgery, there was a significant reduction in pre and post-colony counts among both the mouthwash group patients. However, there was no significant difference in the antimicrobial efficacy between the mouthwashes, suggesting blue®m was equally effective as CHX [25].

Similarly, blue®m was found to be an alternative to CHX in reducing the total microbial load, especially *Porphyromonas gingivalis* load among the patients who had undergone implant surgery [26]. Our results are in accordance with the previous studies, as pre-procedural rinsing with blue®m resulted in lesser microbial colony count in aerosols as compared to CHX, minimizing the extent of microbial contamination. Therefore, the use of blue®m mouthwash as part of routine protective measures can significantly reduce the risk of contamination and transmission of infectious agents, contributing to a safer dental environment for both operator and patient.

Limitations

The above-mentioned study has an observational study design, which is the lowest level of evidence. A small sample size has been considered in this study. Further studies with large sample sizes can substantiate the results of this study.

## Conclusions

This study's findings suggest that there is a reduction in the bacterial load in the aerosols that are emitted during the ultrasonic scaling procedure with the use of oxygen-enriched mouthwash (blue®m) as a pre-procedural rinse when compared with chlorhexidine. This research paves the way to use oxygen-enriched mouthwash as a safe alternative to chlorhexidine as a pre-procedural rinse in routine dental practice.
